# Cathepsins: a new culprit behind abdominal aortic aneurysm

**DOI:** 10.1186/2050-490X-1-5

**Published:** 2013-11-01

**Authors:** Yi Wang, Chaoshu Tang, Yanwen Qin

**Affiliations:** The Key Laboratory of Remodeling-related Cardiovascular Diseases, Beijing An Zhen Hospital, Capital Medical University, Ministry of Education, Beijing Institute of Heart, Lung and Blood Vessel Diseases, Beijing, 100029 China

**Keywords:** Abdominal aortic aneurysm, Extracellular matrix, Cathepsins

## Abstract

Abdominal aortic aneurysm (AAA) is a fatal disease defined as an abdominal aortic diameter of 3.0 cm or more, where the abdominal aorta exceeds the normal diameter by more than 50%. Histopathological changes of AAA mainly include extracellular matrix (ECM) remodeling at the abdominal aorta wall, but there is lack of specific drugs to treat AAA. Recent studies have reported that lysosomal cathepsins could induce vascular remodeling and AAA formation by regulating vascular inflammation, medial smooth muscle cell apoptosis, neovascularization, and protease expression. Thus, cathepsins are expected to become a new therapeutic target for AAA treatment.

## Review

Abdominal aortic aneurysm (AAA) is a fatal disease in which the abdominal aorta diameter is more than 50% its normal diameter, exceeding 3.0 cm [1]. AAA is like a time bomb in the body because the progressive increase in aneurysm size eventually leads to aneurysm rupture and uncontrollable bleeding, resulting in a mortality of 80%. The primary clinical treatments for AAA include open surgical repair and endovascular therapy, but so far there are no specific drugs to prevent or reverse AAA progression. Thus, studying AAA pathogenesis is necessary to explore and identify treatment targets.

## AAA, a common high-risk vascular disease

With the aging of the Chinese population, dietary changes, and new testing methods, the prevalence of AAA has increased dramatically, but there is still no epidemiological data regarding AAA in China. According to published epidemiological data, AAA is the 13th most common fatal disease in the United States. The prevalence of AAA among the US population in those over 65 years old is 8%, and about 25,000 patients undergo AAA repair annually [2–4].

Current AAA treatments include open surgical repair and endovascular therapy, which mainly targets aneurysms greater than 5.5 cm in diameter, or rapidly growing aneurysms that are at high risk of rupture. However, AAA screening suggests that more than 90% of AAAs are small aneurysms less than 5.5 cm in diameter, so limiting the expansion of small-diameter aneurysms has become a priority in the treatment strategy for AAA [5]. Results from animal experiments and clinical trials have suggested that doxycycline, roxithromycin [6], statins [7–9], 2,4-thiazolidinedione [10], β-blockers [11], and angiotensin-converting enzyme inhibitors [12] can suppress AAA progression, but further mechanistic studies and long-term, large-scale randomized controlled trials are required to verify the effects of these drugs.

AAA is a complex disease caused by various factors [13]. It is often seen in older men, and the ratio of male and female patients is about 10:3. Progressive aortic dilatation and atherosclerosis during aging induces the development of AAA. Smoking [14–16] is a major risk factor for AAA, and it may be induced by nicotine in plasma. It is suggested that Chlamydia pneumoniae [17] and cytomegalovirus infections [18] may also be involved in AAA pathogenesis. Though AAA and atherosclerosis share some common environmental and genetic factors, recent studies have demonstrated that the AAA disease process is independent of atherosclerosis [19]. Hypertension promotes AAA formation through artery wall hardening, and there is evidence of genetic predisposition to AAA.

## Vascular remodeling is the pathological basis for AAA

AAA is a chronic degenerative disease that begins with local injuries at the abdominal aorta wall followed by subsequent lesions that affect the entire vessel wall. Histological changes in AAA mainly involve 1) inflammatory cell invasion of the aorta, medial thinning, and adventitial thickening of the aneurysm; 2) decreased elastin level (from 15-33% to 5-8%) and imbalanced elastin/collagen ratio, resulting in aorta elasticity loss and vessel dilation; 3) vascular smooth muscle cell (VSMC) apoptosis in the tunica media that diminishes vessel repair capacity; and 4) inflammation of the aortic adventitia and perivascular tissues, which are accompanied by angiogenesis. All these changes eventually lead to aortic wall remodeling, progressive luminal expansion, and aneurysm rupture [20].

Though the mechanism underlying vascular remodeling during AAA formation has not been fully elucidated, present studies suggest that AAA pathogenesis is associated with inflammatory reactions. Any factors that cause intimal or adventitial injuries can initiate inflammation and immune reactions. Various immune and stromal cells, including macrophages, neutrophils, mast cells, B/T lymphocytes, fibroblasts, and VSMCs, are involved in AAA development [21, 22]. In the early stage of AAA formation, inflammatory cells recruited at abdominal aortic wall release various cytokines, chemokines, and proteases to induce VSMC apoptosis, ECM protein degradation, vascular cell migration, and angiogenesis.

It is now believed that medial VSMC apoptosis is the key to AAA development. VSMCs in AAA tissue show obvious disorganization and an increased apoptotic index. VSMCs play a dual role in ECM metabolism; its affects ECM synthesis by secreting tissue inhibitor of metalloproteinases (TIMP), tropoelastin, and collagen, and it mediates ECM degradation by secreting cytokines to recruit inflammatory cells and elevate matrix metalloproteinase (MMP) synthesis and secretion.

MMP-activated elastin and collagen degradation is suggested to be the direct factor underlying vascular remodeling during AAA development [23]. MMPs can degrade elastin, the major component of ECM, and the degradation products are capable of inducing inflammatory cell infiltration, which in turn increases MMP synthesis through a positive feedback loop and changes aortic wall elasticity, resulting in aortic expansion and aneurysm formation. MMP-2 and MMP-9 play a dominant role in AAA pathogenesis [24].

Though extensive research has been conducted on AAA pathogenesis, the use of anti-inflammatory drugs or MMPs inhibitors to alleviate AAA progression did not yield satisfactory results in clinical trials [25–27]. However, other tissue matrix-degrading enzymes, cathepsins, have received considerable attention for their role in AAA pathogenesis [28].

## Cathepsins, the key factor in vascular remodeling during AAA

Cathepsins are a class of lysosomal proteases that become activated in an acidic environment [29]. Human cathepsins can be assigned into B, C, D, F, G, H, K, L, O, S, V, W, and X subtypes, most of which are cysteine proteases, with the rest being aspartic acid or serine proteases. Cathepsins can be activated by the hydrolysis of an inactive zymogen precursor at the low pH found in the lysosome. AAA risk factors, such as smoking, hypertension, and atherosclerosis, can all cause vascular endothelial cell damage, which increase lysosomal membrane permeability and induce cathepsin secretion. The activity of cathepsins can be modulated by two factors: environmental pH value and the presence of cathepsin endogenous inhibitors (such as cystatin C), which suppress cathepsin activity through binding its target proteins. Studies have suggested [30] that inflammatory factors such as tumor necrosis factor (TNF)-α and interferon (IFN)-γ can induce the secretion of cathepsins from vascular endothelial cells, macrophages, and VSMCs. Nicotine stimulates cathepsin L secretion from pheochromocytoma, and free cholesterol induces cathepsin K release from macrophages by activating the Toll-like receptor and p38 mitogen-activated protein kinase [31]. Oxidized low-density lipoprotein (LDL) stimulates cathepsin S expression and secretion from macrophages [32]. Angiotensin II (AngII)-treated macrophages exhibit increased lysosomal membrane permeability and rupture and induce cathepsin F expression, secretion, and activation [33].

The effects of cathepsins in cardiovascular diseases have raised increasing concern in recent years. Cathepsin expression level and activity are significantly elevated during AAA formation [34, 35]. Immunohistochemistry and western blot demonstrated a lack of cathepsin L in normal vessels but strong positive signals in AAA tissues. Cathepsin L mRNA levels in AAA lesions exceeded that in normal aorta tissue by 22%, and cathepsin H protein levels in AAA lesion was 330% higher than aorta tissue sampled from patients with artery occlusion. Immunofluorescence probes revealed that compared with occluded artery, the activities of cathepsin B, H, L, and S in AAA tissues were increased by 376%, 191%, 223%, and 20%, respectively. Studies also demonstrated that compared with normal vessels, the expression of each cathepsin subtype in AAA tissues was significantly increased. In AAA lesions, luminal endothelial cells mainly expressed cathepsin D, as well as cathepsin B, K, and S, while neovascular endothelial cells, macrophages, and VSMCs could express all cathepsin subtypes. It has been suggested [36] that the expression level of cathepsin S in AAA patient serum is increased by ~30%, and the magnitude of elevation was significantly associated with the size of the AAA. Other reports described that the expression and activity of cathepsin D in AAA patients’ serum and plasma were significantly higher than in healthy controls. In addition, cathepsin expression and activation is often observed in AAA animal models. Immunohistochemistry and immunoblotting conducted in a mouse AAA model (established by 28-d AngII infusion in apolipoprotein [ApoE]-/- mice) showed that [37] normal vessels barely expressed cathepsin S, while AAA tissue had significantly increased cathepsin S expression, which exceeded that in the normal vessel by 90%. Also, the expression levels of cathepsin K, S, and C were 5-fold higher than that in normal vessels. In a 14-d elastase-infused rat AAA model, increased cathepsin C expression was observed at an early stage of AAA [38].

Studies of cathepsin knock-out mice confirmed the essential role of cathepsins in AAA pathogenesis (Table 1). Qin et al. [37] established a cathepsin S/ApoE double knockout mouse model using AngII infusion, and found that the cathepsin S-knockout mice showed significantly reduced AAA incidence (1/8 of that observed in ApoE-/- mice), and a 47% (0.86 mm) decrease in abdominal aortic diameter [7]. The incidence and diameter of AAA in elastin-infused cathepsin K [39] or L [40] knock-out mice were significantly reduced, and these mice also showed decreased T lymphocyte migration, neovascularization, MMP expression, and cathepsin activation. As for elastin-infused cathepsin C-knockout mice, they can hardly form AAA [41]. Conversely, cathepsin C/ApoE double knockout mice infused with AngII exhibited 55% and 50% increases in AAA lesion size and diameter, respectively [38]. *In situ* zymography demonstrated that CaCl_2_-infused AAA mouse showed suppressed elastase activaty, enhanced aorta stability, and reduced vessel damage when treated with serine protease inhibitor A3, a non-specific inhibitor of cathepsin G.

**Table 1:** **Cysteinyl cathepsins and AAA**

Cathepsins	Role in AAA
Cathepsin G inhibition	Suppress elastase activaty, enhance aorta stability, reduce vessel damage
Cathepsin K knockout	Decrease T lymphocyte migration, neovascularization, MMP expression, and cathepsin activation
Cathepsin L knockout	Lower the number of macrophages and decrease T lymphocyte migration, neovascularization, MMP expression, and cathepsin activation
Cathepsin S knockout	Damage elastin integrity, increase collagen accumulation, lower the number of macrophages

The mechanisms and effects of cathepsins in AAA pathogenesis are not been fully understood. Studies have demonstrated [42] that cathepsin S-knockout mice have damaged elastin integrity and increased collagen accumulation at the arterial wall, while small molecule inhibitors of cathepsin S suppressed elastase activity by 80%, suggesting that active cathepsin S could degrade extracellular soluble elastin. Shi et al. [29, 40] found that after knockout of cathepsins S and L, the number of macrophages were lowered through reductions in monocyte chemoattractant protein-1 (MCP-1) and macrophage migration, thereby alleviating inflammation reactions during AAA development. By using AngII to treat macrophages of cathepsin S-knockout mice, Pan [43] demonstrated that cathepsin S could modify inflammatory fibrosis by inducing mitochondrial damage, elevating reactive oxygen species levels and increasing nuclear factor kappa-B (NF- κB) activity. By using the selective cathepsin S selective inhibitor Compound A to treat ozone-exposed neutrophils in the bronchoalveolar lavage fluid, Williams et al. [44] found that inhibiting cathepsin S could reduce interleukin (IL)-6 and TNF-α levels, thus alleviating inflammatory reactions. Qin et al. [37] used the selective cathepsin S Z-FL-COCHO against AngII-induced VSMC apoptosis, and their results suggested that inhibiting cathepsin S decreased apoptosis by down-regulating both the Bax/Bcl-2 ratio and caspase-3 expression. Cathepsin G can induce neovascularization in tumor tissues by activating the transforming growth factor (TGF)-β pathway and enhancing the expression of vascular endothelial growth factor (VEGF) and MCP-1 [45]. It can also increase MMP expression by promoting the conversion of MMP-1 zymogen into active MMP-1 [46]. Cathepsin G [47] can promote conversion of Ang I to Ang II, and it is also capable of generating Ang II directly from angiotensinogen. Daugherty et al. [48] demonstrated that apoE^-/-^ mice infused with Ang II had increased plasma concentrations and pronounced abdominal aortic aneurysms.

## Conclusions

In summary, increased cathepsin expression levels and reduced levels of their inhibitors in human AAA lesions suggest a role of these proteases in AAAs. Reduced AAA size and incidence in cathepsin-deficient mice but enlarged aortic size in the absence of their inhibitors, demonstrates that these proteases directly participate in AAA pathogenesis. Although selective inhibitors for several major AAA cathepsins are available, none have been tested in experimental AAAs or in humans. Serum biomarkers have proved useful in assessing the risk associated with several diseases. Plasma cathepsin S levels are significantly higher in AAA patients than in age-matched healthy controls [36]. Serum cathepsin S and hs-CRP are independently correlated with the maximum diameter of the abdominal aorta. Combined serum cathepsin S and hs-CRP levels are better at indirectly predicting the maximum diameter and the degree of inflammatory response in AAA lesions in the clinical setting [49]. This evidence suggests that cathepsins may be used as a diagnostic indicator of AAA.

While studies have shown that cathepsins are directly involved in AAA pathogenesis, the relationships between AAA and different cathepsin subtypes, the interactions between cathepsins and other risk factors for AAA and the effects of cathepsins in different stages of AAA development remain to be elucidated. Although the study of cathepsin inhibitors on treating AAA is limited to pre-clinical research, lysosomal cathepsins as a diagnosis tool and treatment target for halting AAA development are of particular clinical significance and should be paid considerable attention.
